# Multisystem Light Chain Amyloidosis: Diagnostic and Therapeutic Challenges

**DOI:** 10.7759/cureus.91334

**Published:** 2025-08-31

**Authors:** Htet Zaw Lin, Kay Kay Khaing Ko, Thiraviyam Kandaswamy, Vui H Chong, Jackson Tan

**Affiliations:** 1 Department of Nephrology, Raja Isteri Pengiran Anak Saleha Hospital, Bandar Seri Begawan, BRN; 2 Department of Cardiology, Raja Isteri Pengiran Anak Saleha Hospital, Bandar Seri Begawan, BRN; 3 Department of Medicine, Raja Isteri Pengiran Anak Saleha Hospital, Bandar Seri Begawan, BRN

**Keywords:** amyloid light chain (al) amyloidosis, gastrointestinal amyloid, renal amyloid, restrictive cardiomyopathy, secondary membranous nephropathy

## Abstract

Amyloid light (AL) chain amyloidosis is a rare, progressive multisystem disorder caused by the deposition of misfolded monoclonal light chains, which are produced by clonal plasma cells and commonly affect the kidneys and heart. The overall prognosis depends on the extent of organ involvement and timely diagnosis. A previously healthy 57-year-old man presented with a two-month history of progressive lower limb edema and exertional dyspnea. Initial investigations revealed elevated troponin, hypoalbuminemia, and nephrotic-range proteinuria. Coronary angiography was normal, but echocardiography showed restrictive cardiomyopathy. Renal biopsy after light and immunofluorescence microscopy was reported as membranous nephropathy. However, there was no response to proteinuria to sacubitril/valsartan, sodium-glucose co-transporter 2 (SGL2) inhibitor, and steroid therapy. The treatment was escalated with additional immunosuppressive treatments, including cyclosporine and rituximab. Subsequently, Congo red staining of the renal biopsy confirmed a diagnosis of amyloidosis. Further, a hematologic workup showed marked lambda (λ) light chain predominance with a suppressed kappa (κ)/λ ratio, and fluorescence in situ hybridization (FISH) analysis revealed a t (11;14) translocation, confirming AL amyloidosis. The patient was treated with daratumumab, bortezomib, cyclophosphamide, and dexamethasone (Dara-VCD), which was complicated by cytomegalovirus (CMV) gastritis. Despite receiving three cycles of therapy, the patient’s condition deteriorated due to advanced cardiac failure, autonomic neuropathy, and a severe CMV infection. He ultimately succumbed to the illness 18 months after the initial presentation. This case highlights the diagnostic complexity of AL amyloidosis and the importance of early recognition. Clinicians should consider AL amyloidosis in patients with unexplained nephrotic syndrome and heart failure.

## Introduction

Amyloid light-chain (AL) amyloidosis is a rare, progressive, and often fatal systemic disorder caused by the misfolding of immunoglobulin light chains secreted by clonal plasma cells. These misfolded proteins aggregate into amyloid fibrils that deposit in various organs, most commonly the heart and kidneys, resulting in progressive organ dysfunction [1]. The prognosis varies significantly and is primarily influenced by the extent of organ involvement (particularly cardiac involvement), the severity of the underlying plasma cell dyscrasia, and the patient's overall response to therapy. Early clinical recognition is critical for a timely diagnosis and management. Without treatment, the median survival is only six to 12 months, especially in patients with advanced cardiac involvement [1,2]. The treatment approach for AL amyloidosis differs significantly from that of transthyretin amyloid cardiomyopathy (ATTR-CM). Chi et al. reported on the effects of adding sodium-glucose cotransporter 2 (SGLT2) inhibitors to tafamidis therapy in patients with ATTR-CM. These agents were well tolerated and associated with improved fluid balance and symptom relief, suggesting that SGLT2 can complement tafamidis in routine clinical practice [3]. In a separate analysis, the same group also evaluated the use of mineralocorticoid receptor antagonists (MRAs), such as spironolactone or eplerenone, in tafamidis-treated patients. Their findings highlighted potential symptomatic benefits and improved cardiac functional status, while also noting risks such as electrolyte disturbances [4].

The global incidence of AL amyloidosis is estimated at approximately one case per 100,000 person-years, with significantly fewer cases reported from Southeast and South Asia [5-10]. Due to its rarity and overlapping symptoms with other more prevalent diseases, AL amyloidosis is often misdiagnosed, particularly in resource-limited settings. The clinical manifestations are diverse and frequently nonspecific, contributing to significant diagnostic delays. Common presentations include nephrotic-range proteinuria, unexplained heart failure with preserved ejection fraction (HFpEF), orthostatic hypotension, and peripheral neuropathy [11-13]. Diagnostic confirmation relies heavily on Congo red staining, which reveals characteristic apple-green birefringence under polarized light [14]. While mass spectrometry and electron microscopy are considered gold standards for amyloid typing [15], these modalities may be unavailable in low-resource environments. Renal biopsy remains essential in patients presenting with unexplained nephrotic syndrome, and timely recognition is key to improving outcomes. 

Here, we present the case of a previously healthy 57-year-old man who developed multiorgan AL amyloidosis with delayed diagnosis following an initial misclassification as membranous nephropathy. This case highlights the challenges of early recognition, the prognostic utility of beta-2 microglobulin, and evolving therapeutic options.

## Case presentation

A 57-year-old previously fit and active police officer presented to the emergency department with two months of progressive bilateral lower limb swelling, intermittent exertional dyspnea, and orthopnea. He reported no significant reduction in urine output or other urinary symptoms. His past medical history was unremarkable.

**Figure 1:**
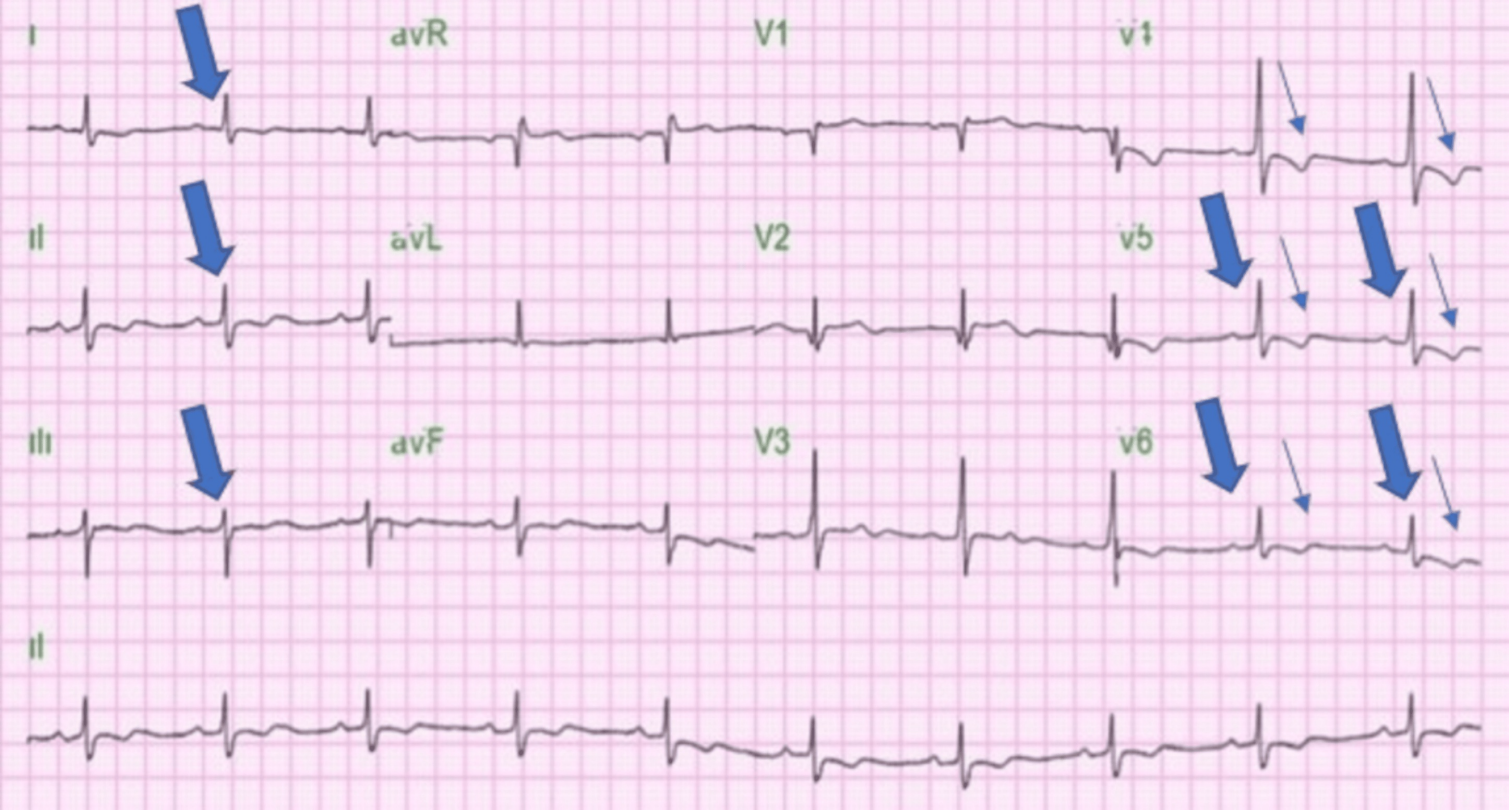
Electrocardiogram of the patient showing low voltage QRS in both the precordial lead (<10mm) and limb leads(<5mm) (thick arrows), and nonspecific ST and T wave changes (thin arrows)

On admission, he was hypertensive and showed signs of volume overload. The initial clinical impression was non-ST elevation myocardial infarction (NSTEMI) due to elevated troponin I (up to 1447.5 ng/L; normal valve: 0.0-34.1ng/L) complicated by heart failure and grade II hypertension. He was admitted to the cardiology service and was on sacubitril/valsartan, SGL2 inhibitor, and intravenous furosemide. Chest X-ray was reported as normal cardiac size, and electrocardiogram showed low voltage QRS in both precordial leads (<10mm) and limb leads(<5mm), and non-specific ST and T wave changes (Figure 1).

**Figure 2:**
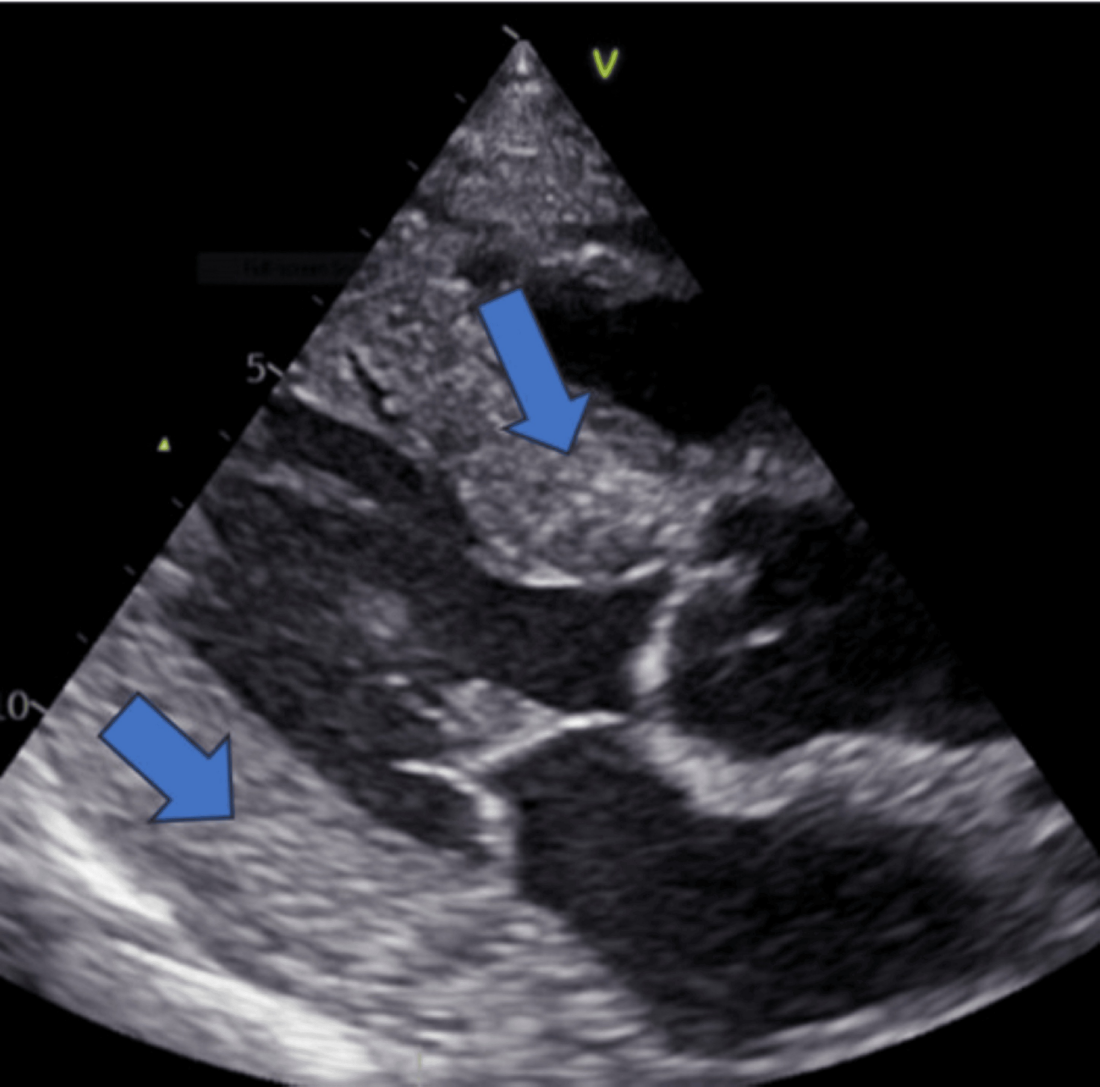
The echocardiogram showed biventricular hypertrophy with sparkling texture of myocardium (blue arrows).

Echocardiography done by the echo technician was reported as showing ventricular hypertrophy with preserved ejection fraction. In view of the high troponin I, the patient was transferred to the tertiary cardiac center, and coronary angiography revealed normal coronary arteries. At this point, a repeat echocardiography by the resident cardiologist revised the diagnosis to restrictive cardiomyopathy. The echocardiography also showed a scattered sparkling texture of the myocardium (Figure 2).

Further investigations revealed nephrotic-range proteinuria with a urine protein-creatinine ratio (uPCR) of approximately 2,958.9 mg/mmol, hypoalbuminemia (21 g/L; normal: 35-50 g/L) with an estimated glomerular filtration rate of 70.7 ml/min/1.73 m². Subsequently, nephrology input was sought due to nephrotic-range proteinuria and persistent fluid overload. The ultrasound scan of the kidneys was normal apart from slightly enlarged kidneys. At the time, a small supraclavicular lymph node was incidentally found, raising concern for a secondary cause of nephrotic syndrome such as amyloidosis or lymphoma. Serum C3 and C4 were within normal limits and negative for all autoimmune screenings, excluding complement-mediated glomerulopathies such as lupus nephritis or C3 glomerulopathy. Serum protein electrophoresis was also less than 1 gram per day, and immunofixation showed IgG kappa (κ) by anti-IgG, anti-IgA, anti-IgM, anti-K, and anti-lambda (λ). He underwent a renal biopsy and was discharged with outpatient follow-up.

Renal biopsy initially revealed membranous nephropathy, with 13 glomeruli sampled (one globally sclerosed). There were no crescents or necrosis. H&E sections showed mesangial expansion, and silver staining demonstrated glomerular "spikes." Immunofluorescence was positive for IgG and IgM, with patchy IgA staining; C3d and fibrinogen were negative. Electron microscopy, the gold standard for diagnosing membranous, was not available in the country. Additionally, Congo red staining was also not the routine used in our histopathology services. Based on these findings, a diagnosis of membranous nephropathy was made, and the patient was initiated on immunosuppressive therapy with prednisolone. After two months of therapy, proteinuria persisted despite treatment with sacubitril/valsartan, an SGLT2 inhibitor, and corticosteroid, and it was planned to initiate rituximab therapy for the treatment of steroid-nonresponsive membranous nephropathy.

**Figure 3:**
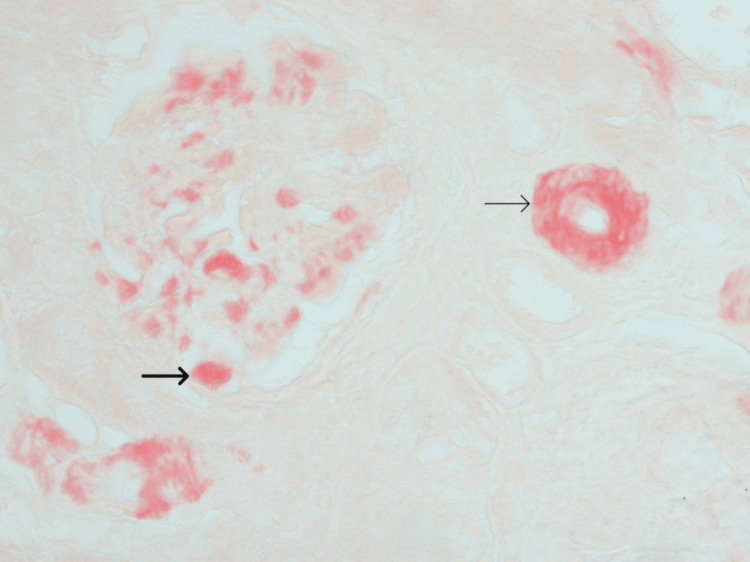
Congo red staining showed that strong orangeophilic staining was present within the glomeruli (thick arrow) and arteriolar wall (thin arrow).

**Figure 4:**
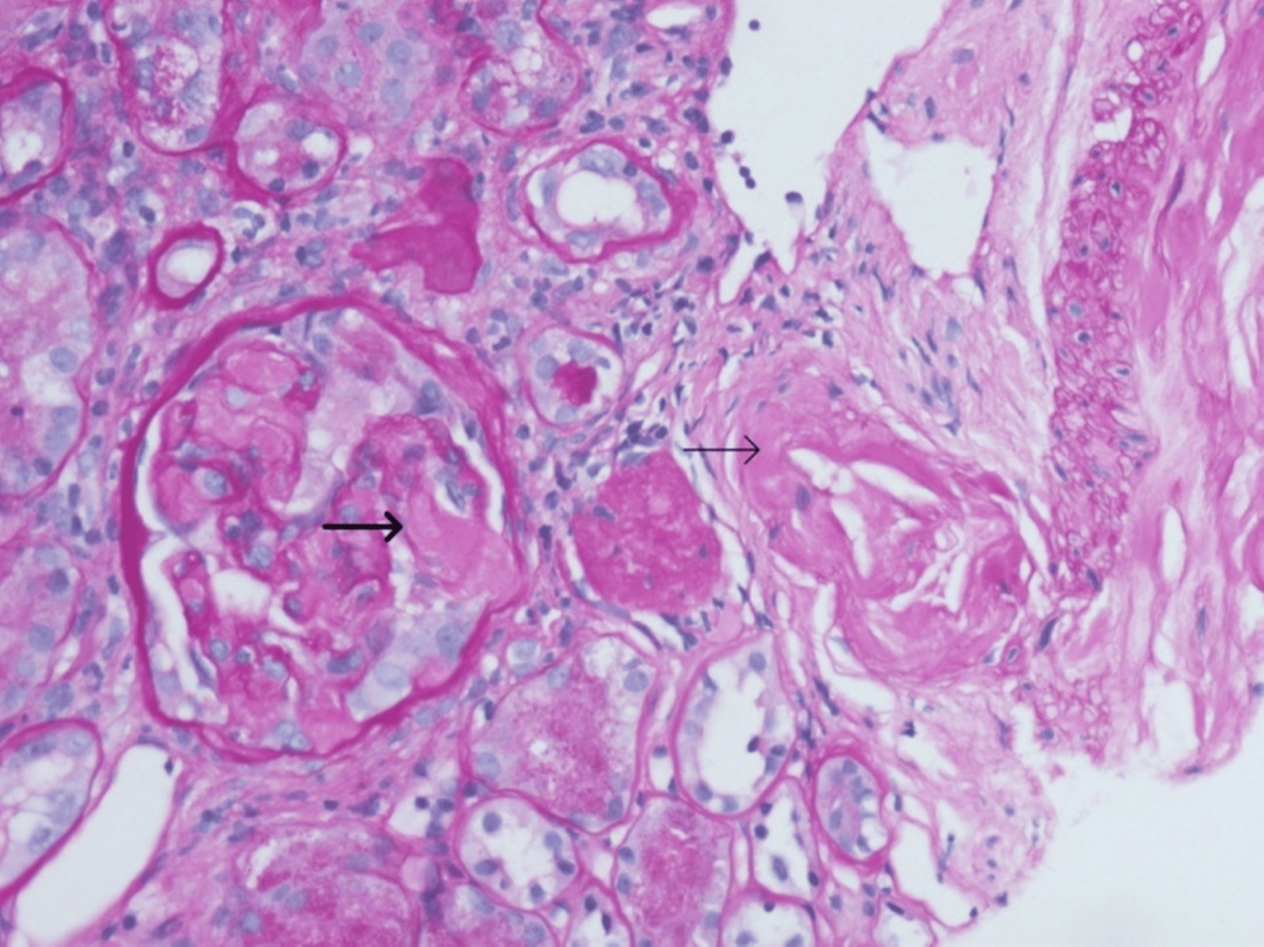
Pale staining mesangial regions within glomeruli (thick arrow) and arteriolar wall (thin arrow)

In the meantime, during his cardiac follow-up, a request for Congo red staining of the renal biopsy was made. This demonstrated amyloid deposition in the glomeruli and vascular intima, leading to a revision of the diagnosis to renal amyloidosis (Figures 3, 4).

At the planned admission for rituximab therapy, in light of the revised diagnosis of renal amyloidosis, the patient was referred to the hematology service for evaluation of a possible plasma cell dyscrasia or lymphoproliferative disorder. The left supraclavicular lymph node biopsy was negative for malignancy. As part of the systemic work-up, a cardiac magnetic resonance imaging was done, and this showed features suggestive of cardiac amyloidosis. A bone marrow examination was planned, but the patient declined any further investigations and interventions.

He was subsequently readmitted three (April 2024) and nine months (October 2024) later with recurrent episodes of presyncope and dizziness. On each occasion, he was noted to be in heart failure with hypotensive and bradycardic responses to fluid resuscitation. Due to persistent hypotension and bradycardia, the patient was deemed unsuitable for further angiotensin receptor-neprilysin inhibitor (ARNI) therapy. A likely diagnosis of autonomic neuropathy was considered, and during the latest admission, the patient agreed to proceed with further investigations.

Further evaluation revealed a monoclonal light chain pattern characterized by mildly elevated (20.6 mg/L) or normal κ light chains (3.30-19.4 mg/L), significantly elevated λ light chains (peak 412 mg/L; normal: 5.71-26.3 mg/L), and a persistently suppressed free light chain ratio (0.088-0.246; normal: 0.26-1.65), strongly suggestive of monoclonal λ chain production (λ-restricted clone). Urine electrophoresis detected free λ light chains, consistent with Bence Jones proteinuria. Additionally, a bone marrow aspiration and trephine biopsy showed mild hypercellularity with trilineage hematopoiesis and a myeloid-to-erythroid ratio of 5:1. No abnormal infiltrates or fibrosis were seen. Congo red staining of the marrow was negative, and cytogenetics revealed a normal male karyotype (46, XY). However, fluorescence in situ hybridization (FISH) analysis was positive for t (11;14), a finding associated with AL amyloidosis. Hereditary transthyretin amyloidosis was initially considered in the differential diagnosis but was later excluded.

By this time, the patient had demonstrated immunoparesis and hypogammaglobulinemia, with a gradual decline across all immunoglobulin classes, particularly IgG and IgA. Additionally, beta-2 microglobulin was markedly elevated (up to 8.45 mg/L; normal range: 0.97-2.00 mg/L), indicating severe tumor burden and poor prognosis in AL amyloidosis.

Following this, the patient was initiated on subcutaneous daratumumab, bortezomib (Velcade), cyclophosphamide, and dexamethasone (Dara-VCD). Unfortunately, he developed dysphagia after two cycles of chemotherapy, and an upper gastrointestinal endoscopy showed extensive nodular, friable, raised masses throughout the gastric fundus and body. Biopsies were positive for cytomegalovirus (CMV)-related acute-on-chronic gastritis. Congo staining was also positive. He was treated for CMV infection (14 days of intravenous ganciclovir followed by six weeks of oral ganciclovir), and a repeat biopsy several weeks later showed resolution of CMV.

Despite three cycles of chemotherapy and supportive therapy, the patient succumbed to this illness 18 months after the initial presentation with sudden cardiac arrest due to advanced cardiac failure and autonomic abnormalities.

## Discussion

AL amyloidosis is a rare disease, with an incidence of approximately one case per 100,000 person-years in the United States of America [5]. To date, there have only been a few cases reported from the Southeast Asia and South Asia region (Table 1).

**Table 1:** Cases of AL amyloidosis reported from Southeast Asia and South Asia regions AL amyloidosis: amyloid light-chain amyloidosis; ANA: antinuclear antibody; ATTR: transthyretin amyloidosis; BMI: body mass index; CMV: cytomegalovirus; DLBCL: diffuse large B-cell lymphoma; ECG: electrocardiogram; ECHO: echocardiogram; EF: ejection fraction; eGFR: estimated glomerular filtration rate; Hb: hemoglobin; hs-cTnT: high-sensitivity cardiac troponin T; IPF: immature platelet fraction; κ/λ ratio: kappa to lambda light chain ratio; LDH: lactate dehydrogenase; NT-proBNP: N-terminal pro B-type natriuretic peptide; PCR: polymerase chain reaction; PET/CT: positron emission tomography/computed tomography; RCHOP: rituximab, cyclophosphamide, doxorubicin, vincristine (Oncovin), and prednisolone; R-GemOx: rituximab, gemcitabine, and oxaliplatin Source: [3-7]

Cases and reference	Hu et al. [6]	Sukrisman et al. [7]	Sangluthong et al. [8]	Islam et al. [9]	Duong et al. [10]
Origin	Malaysia	Indonesia	Thailand	Bangladesh	Vietnam
Age and gender	66 years, female	55 years, female	69 years, female	50 years, female	47 years, male
Medial problems	Not mentioned	Inflammatory bowel disease, Enlarged neck lymph nodes (follicular stage 3B and DLBCL) on RCHOP therapy	Diabetes mellitus, hypertension, hypothyroidism, chronic kidney disease stage IV	Non-diabetic, normotensive, no heart disease	Non-diabetic, normotensive, no heart disease
Symptoms	Bilateral upper and lower limbs asthenia x years, loss of weight (28Kg)	Chronic diarrhea and fever; a colonic biopsy revealed recurrent DLCBL and repeated V6 cycles of RCHOP. One year later, the patient presented with recurrent fever and weight loss and was diagnosed with acute CMV infection; therefore, treatment of acute CMV infection and the chemotherapy regimen were changed to R-GemOx. One year later, the patient presented again with severe abdominal pain and a persistent fever.	Anasarca for a week	Pain in multiple joints for two years, and a skin lesion in different parts of the body for one year	Severe dizziness and fainting episodes for a week, weight loss of 20 kg
Clinical features	No organomegaly; lower limbs had moderate edema, small hand muscle wasting, bilateral foot drops, and impaired sensory column in both upper and lower limbs.	Anemia	Anasarca, no organomegaly	Skin lesions were raised, fluid-filled, around her mouth, upper lip, around her eyes, around her anus, painless, not itchy, no abnormal sensation, macroglossia	Heart rate: 12-15 beats per minute, blood pressure: 60/40mmHg, BMI 14.38kg/m^2^
Investigations	Hypoalbuminemia (24.8 g/dL), 1 g proteinuria per day, normal immunology assays, low complement C3, and low immunoglobulin IgG levels	Severe anemia (Hb: 9.7mg/dl) and neutropenia, normal kidney function, NT-proBNP, 7,664 ng/l, hs-cTnT, 22.4 pg/ml, urea (164 mg/dl), creatinine (3.67 mg/dl), IPF (9%), and LDH (546 IU/l)_> diagnosed as heart failure	3.5 grams per day 4+ proteinuria in urinalysis, hypoalbuminemia (2.6 g%) Subclinical hypothyroidism ANA positive (1:2560) eGFR: 23 ml/m²/min	Normal urine RME, 24-hour urinary protein was 0.17 gm, serum albumin- 37 gm/L	eGFR 34 ml/min, hs-cTnT of 252 pg/mL, and heart failure with NTproBNP of 13259 pg/mL.
Immunofixation	Not mentioned	Not mentioned	κ 122 mg/l, λ55.9 mg/l ratio: 2.1	Serum protein electrophoresis shows the alpha-2 region raised, normal immunofixation study	Slight increase of free κ(60.5 mg/L) and free λ (71.5 mg/L); however, the κ/λ ratio is normal (0.84).
ECG	Low voltage in limb leads	Not mentioned	Not mentioned	Not mentioned	Complete heart block
ECHO	Increased interventricular septum and left ventricular wall thickness with normal echo; columnar papillaries were thickened	Normal heart structure and left ventricular EF of 72%.	Left ventricular EF of 66%, Granular sparking pattern at the interventricular septum with moderate pericardial effusion	Left ventricular EF of 65%.	Very thick and bright heart muscle walls, with preserved left ventricular systolic function (EF 58%)
Ultrasound	Normal kidney sizes	Not mentioned	Increase the echo of the renal parenchyma of the bilateral kidneys; normal kidney sizes (10.7cm)	Increase the echo of the renal parenchyma of the bilateral kidneys.; normal kidney sizes (9.7cm)	
PET/CT	Hypertrophy with pericardial effusion, the density of subcutaneous fat all over the body was raised; the metabolism of fluorodeoxyglucose was raised in the above-mentioned focus areas, and the distal end of the rectum	Lymph node enlargement in the lower-right, mid-, and upper-left abdominal areas	Not mentioned	Not mentioned	Cardiac magnetic resonance shows diffuse late gadolinium enhancement in both ventricles and atria with a significant wall thickness of #18 mm, minimal pericardial effusion. It preserved left ventricular function with an EF of 60%.
Bone marrow biopsy	Active hyperplasia and normal plasma cells	Bone marrow puncture results indicated hypercellular bone marrow infiltration by lymphoma and malignant large lymphoid cells, consistent with metastasis of DLBCL to the bone marrow	Cell: fat=30:70, myeloid: erythroid =2:1, no fibrosis, no granuloma	Not mentioned	
Biopsy	The part of the blood vessel walls of the renal interstitium was stained by Congo red; some of the amyloid is indicated by arrows (renal).	Congo red and periodic acid-Schiff staining confirmed the presence of amyloid materials in the lamina propria, diagnosing gastric amyloidosis (gastric).	Congo red staining showed positive apple green birefringence (abdominal fat pad).	Skin biopsy and histopathology from the lower lip show epidermis hyperkeratosis and a few follicular plugs containing demodex folliculorum. The superficial and upper part of the dermis revealed organophilic deposits of amyloid-like material. It shows apple green birefringence in polarized microscopy. A diagnosis of AL amyloidosis	A biopsy of the abdominal fat, as well as a tongue biopsy, was all negative for Congo red spot. hereditary ATTR subtype, the patient’s blood was sent to Green Cross Laboratories (South Korea), using the PCR and sequencing (total 4 exons) method. The result shows heterozygous for c.209G>T (p.Ser70Ile), which is known to be hereditary ATTR.
Outcome	Not mentioned	Died within one year after diagnosis	Not mentioned	Not mentioned	Not mentioned

Most of the cases were associated with plasma cell dyscrasia or monoclonal gammopathy of undetermined significance (MGUS) [11,12]. The median diagnostic delay was six to 12 months from the symptom onset, and AL amyloidosis was usually misdiagnosed as heart failure of preserved ejection fraction (HFpEF), nephrotic syndrome of unclear cause, and chronic inflammatory diseases. The rarity of AL amyloidosis combined with its nonspecific symptoms often results in substantial diagnostic delay, which negatively impacts prognosis [13]. Cardiac amyloidosis is not a simple infiltrative cardiomyopathy: direct toxicity of abnormal precursor proteins and other circulating factors contribute to myocardial dysfunction [16]. Mussinelli et al. reported that quantitative measurements of QRS voltages showed that all indices were depressed in patients with cardiac involvement, confirming the higher prevalence of low QRS voltage in cardiac AL amyloidosis (Sokolow-Lyon index: cardiac AL 7.0 mm (4.0-11.0) vs. non-cardiac AL 14.5 mm (10.0-18.4); p-value < 0.001; peripheral QRS amplitude: cardiac AL 24.0 mm (18.9-31.3) vs. non-cardiac AL 33.7 mm (26.1-42.5); p-value < 0.001) [17].

AL amyloidosis typically involves multiple organs and is rarely confined to a single system. The most common organs affected are the heart and kidneys, but other organs, including the liver, soft tissues, including the skin, the gastrointestinal tract, and the nervous system, can also be affected [18]. In our case, there were cardiac, renal, and gastrointestinal involvements. For the diagnosis of other organ involvement, skin and rectal biopsies can be easily carried out. However, these may not be required if the diagnosis, as in our case, has been established.

Awareness and early clinical recognition are critical for timely diagnosis and management, as they will impact outcomes [1]. Measuring circulating free κ and λ light chains is more sensitive in detecting monoclonal light chains in AL amyloidosis [19]. The diagnostic hallmark of amyloid deposits is their apple green birefringence on Congo staining, viewed on birefringent polarization microscopy, typically from abdominal fat pad, kidney, or bone marrow [14]. Vrana et al. reported that the gold standard for amyloid typing is mass spectrometry-based proteomic analysis, with high sensitivity (98%) and specificity (98%) [15]. Bone marrow biopsy can assess clonal plasma cell burden and confirm plasma cell dyscrasia producing amyloidogenic light chains [20].

Achieving a complete hematologic response is associated with significantly improved survival. High-dose melphalan with autologous stem cell transplant offers long-term remission in selected patients with good performance status [21]. Nowadays, newer therapies, especially proteasome inhibitors (e.g., bortezomib) and anti-CD38 antibodies (e.g., daratumumab), have improved outcomes in patients ineligible for bone marrow transplants [22]. Findings from the ANDROMEDA TRIAL, a Phase III, international, multi-center, randomized clinical trial, compared the efficacy of Dara-VCD to VCD alone in patients with newly diagnosed AL amyloidosis. The trial demonstrated that there was a 59.5% hematological complete response (CR) in the Dara-VCD group vs. a 19% hematological CR in the VCD alone group. Additionally, there was improvement in major organ deterioration progression-free survival (PFS) (hazard ratio 0.4), improved overall survival rate (hazard ratio 0.6), and achievement of complete cardiac response in 40% of patients treated with Dara-VCD [23]. Other studies also showed that prognosis in AL amyloidosis was strongly influenced by the extent of organ involvement, particularly cardiac, and the achievement of hematologic response (Table 2) [24-26].

**Table 2:** Survival estimates of amyloid light (AL) chain amyloidosis Source: [18-20]

Risk group	Median overall survival
No cardiac involvement	>5-7 years
Mild cardiac involvement	2-4 years
Advanced cardiac involvement	6-12 months
Hematological complete response	Up to 10+ years

Despite multiple investigative clues pointing toward the diagnosis, there was a delay in establishing the diagnosis due to system-related factors. These included miscommunication between different clinical services and a lack of timely consideration and requests for appropriate investigations, specifically the Congo red staining, which was not performed. In addition, patient-related factors contributed to the delay. The patient declined further investigations initially, which hindered timely diagnosis. Even at the first presentation, there was a subtle cardiac clue, a scattered sparkling texture of the myocardium, that suggested cardiac amyloidosis, though it was not the classic ‘starry sky’ appearance typically seen in this condition. 

Beta-2 microglobulin is an established prognostic marker in AL amyloidosis. Elevated levels of beta-2 microglobulin correlate with increased disease burden and inferior survival outcomes. It is especially significant in revised staging systems, such as the Mayo 2012 staging, which incorporates NT-proBNP, cardiac troponin T, and difference in free light chains (dFLC) along with beta-2 microglobulin. In a revised prognostic model, Beta-2 microglobulin >2.5 mg/L was identified as an independent predictor of poor prognosis. In this case, beta-2 microglobulin was markedly elevated (up to 8.45 mg/L; normal range: 0.97-2.00 mg/L), which is the independent predictor of poor prognosis, and high dFLC values (>100 mg/L) on initial presentation suggested significant disease burden [27].

Although the patient achieved a very good partial response after three cycles of Dara-VCD, there was no corresponding organ response (Table 3). Ultimately, the patient succumbed to the illness 18 months after the initial presentation and three months following the first cycle of Dara-VCD, due to advanced heart failure, autonomic neuropathy, and infection [27].

**Table 3:** Investigation summary before and after the treatment with Dara-VCD Dara-VCD: daratumumab, bortezomib, cyclophosphamide, and dexamethasone

Date	2023	4/1/2024	21/1/2024	24/10/24	28/10/2024	4/12/2024	6/1/25: After the first cycle of Dara-VCD	1/2/25: After the second cycle of Dara-VCD	25/2/25: after the third cycle of Dara-VCD
Urine protein: Creatinine ratio	800-1000	2255.1	2247.7	2320.7	1864.3	1864.3	2611.6	2958.2	
Troponin I (0.0-34.1ng/ml)	982.5	776.1	1495.2	909.1	670	685.4	300	807.9	980
NT-pro brain natriuretic peptide (NT-proBNP) 90-124pg/ml)	3578	2894	7217	9575	12798	13170	33101	13962	21437
Alkaline phosphatase (ALP) (40-150 U/L)	150	141	147	165	156	205	183	258	253
Serum protein electrophoresis	<1 g/day	<1g/day	<1 g/day		<1g/day	<1g/day	<1g/day	<1 g/day	
Immunophoresis	Mix of IgG kappa and IgG lambda	Mix of IgG kappa and IgG lambda	Free lambda		Free light chain	Free light chain	Ig G kappa	Ig G kappa	
Beta-2 microglobulin (0.97-2mg/L)		3.79(first time) 4.74 (2^nd^ time)		8.45					
Serum kappa light chain (3.30-19.4mg/L)	20.6	38.7	38.7		31.5	36.4	17.3	12.6	14.1
Serum lambda light chain (5.71-26.3mg/L)	177	346	346		340	412	70.3	19.9	25.1
Free light chain ratio	0.116	0.112	0.112		0.093	0.088	0.246	0.633	0.562
Difference in free light chain (dFLC)	156.4	307.3	307.3		308.5	376.6	53	7.3	11.0
Urine electrophoresis						5.81 free lambda light chain			

Although advances in treatment, including daratumumab and bortezomib-based regimens, have improved outcomes, timely diagnosis remains crucial. In this case, FISH analysis was positive for t(11;14). Alvanidis et al. stated that venetoclax, a selective BCL-2 inhibitor, appears to be a promising therapy for patients with relapsed/refractory AL amyloidosis, particularly in patients with the t(11;14) translocation and plasma cell leukemia. However, optimal dosing, combination regimens, and their role in patients without the translocation require further investigation [28].

## Conclusions

This case illustrates the aggressive course of the disease due to the severity of cardiac and renal involvement upon initial presentation. The patient's clinical course underscores the importance of maintaining a high index of suspicion in patients presenting with nephrotic-range proteinuria, unexplained heart failure, or multisystem involvement. Early recognition and timely diagnosis are crucial, as delayed treatment significantly compromises outcomes, particularly in those with cardiac involvement.

## References

[1] Merlini G, Bellotti V (2003). Molecular mechanisms of amyloidosis. N Engl J Med.

[2] Palladini G, Dispenzieri A, Gertz MA (2012). New criteria for response to treatment in immunoglobulin light chain amyloidosis based on free light chain measurement and cardiac biomarkers: impact on survival outcomes. J Clin Oncol.

[3] Chi KY, Varrias D, Borkowski P (2025). Sodium-glucose cotransporter 2 inhibitors in tafamidis-treated transthyretin amyloid cardiomyopathy: a contemporary real-world analysis. JACC Heart Fail.

[4] Chi K-Y, Witteles RM, Cheng RK (2024). Benefits and risks associated with mineralocorticoid receptor antagonists in tafamidis-treated transthyretin amyloid cardiomyopathy: a real-world analysis. Circulation.

[5] Kyle RA, Larson DR, Kurtin PJ (2019). Incidence of AL amyloidosis in Olmsted County, Minnesota, 1990 through 2015. Mayo Clin Proc.

[6] Hu HQ, Wang SC, Cao X, Cao BZ (2010). Primary systemic amyloidosis: a case report and review of clinical features. IIUM Med J Malaysia.

[7] Sukrisman L, Makmun D, Krisnuhoni E (2024). Systemic amyloidosis following inflammatory bowel disease, follicular lymphoma, and diffuse large B-cell lymphoma: a case report. Med J Indones.

[8] Sangluthong L, Insiripong S, Junmuenwai W (2023). Amyloidosis presenting with edema and heavy proteinuria: a case report. Chulalongkorn Med J.

[9] Islam MS, Ahmed QMU, Masud AHM, Uddin MA (2024). Amyloidosis - a rare case report in Bangladesh. Med Res Chron.

[10] Duong TA, Nguyen NTL, Le MH (2022). A case report of the first hereditary transthyretin cardiac amyloidosis diagnosed in Vietnam. Tap Chi Nghien Cuu Y Hoc.

[11] Kyle RA, Gertz MA (1995). Primary systemic amyloidosis: clinical and laboratory features in 474 cases. Semin Hematol.

[12] Quock TP, Yan T, Chang E, Guthrie S, Broder MS (2018). Epidemiology of AL amyloidosis: a real-world study using US claims data. Blood Adv.

[13] Wechalekar AD, Gillmore JD, Hawkins PN (2016). Systemic amyloidosis. Lancet.

[14] Pepys MB (2006). Amyloidosis. Annu Rev Med.

[15] Vrana JA, Gamez JD, Madden BJ, Theis JD, Bergen HR 3rd, Dogan A (2009). Classification of amyloidosis by laser microdissection and mass spectrometry-based proteomic analysis in clinical biopsy specimens. Blood.

[16] Brenner DA, Jain M, Pimentel DR (2004). Human amyloidogenic light chains directly impair cardiomyocyte function through an increase in cellular oxidant stress. Circ Res.

[17] Kim E, Birnbaum Y (2013). Acute coronary syndromes presenting with transient diffuse ST segment depression and ST segment elevation in lead aVR not caused by "acute left main coronary artery occlusion": description of two cases. Ann Noninvasive Electrocardiol.

[18] Sabinot A, Ghetti G, Pradelli L, Bellucci S, Lausi A, Palladini G (2023). State-of-the-art review on AL amyloidosis in Western Countries: epidemiology, health economics, risk assessment and therapeutic management of a rare disease. Blood Rev.

[19] Rao M, Lamont JL, Chan J (2012). Serum Free Light Chain Analysis for the Diagnosis, Management, and Prognosis of Plasma Cell Dyscrasias: Future Research Needs: Identification of Future Research Needs From Comparative Effectiveness Review No. 73 [Internet]. Blood.

[20] Rajkumar SV, Lacy MQ, Kyle RA (2007). Monoclonal gammopathy of undetermined significance and smoldering multiple myeloma. Blood Rev.

[21] Skinner M, Sanchorawala V, Seldin DC (2004). High-dose melphalan and autologous stem-cell transplantation in patients with AL amyloidosis: an 8-year study. Ann Intern Med.

[22] Kastritis E, Palladini G, Minnema MC (2021). Daratumumab-based treatment for immunoglobulin light-chain amyloidosis. N Engl J Med.

[23] Palladini G, Kastritis E, Maurer MS (2020). Daratumumab plus CyBorD for patients with newly diagnosed AL amyloidosis: safety run-in results of ANDROMEDA. Blood.

[24] Dispenzieri A, Gertz MA, Kyle RA (2004). Serum cardiac troponins and N-terminal pro-brain natriuretic peptide: a staging system for primary systemic amyloidosis. J Clin Oncol.

[25] Kumar S, Dispenzieri A, Lacy MQ (2012). Revised prognostic staging system for light chain amyloidosis incorporating cardiac biomarkers and serum free light chain measurements. J Clin Oncol.

[26] Heitink-Pollé KM, Nijsten J, Boonacker CW, de Haas M, Bruin MC (2014). Clinical and laboratory predictors of chronic immune thrombocytopenia in children: a systematic review and meta-analysis. Blood.

[27] Palladini G, Milani P, Merlini G (2020). Management of AL amyloidosis in 2020. Hematology Am Soc Hematol Educ Program.

[28] Alvanidis G, Kotsos D, Frouzaki C, Fola A, Hatjiharissi E (2025). The potential role of BCL-2 inhibition in amyloidosis and plasma cell leukemia. Front Oncol.

